# Innate and acquired tolerance to bitter stimuli in mice

**DOI:** 10.1371/journal.pone.0210032

**Published:** 2018-12-31

**Authors:** Emi Mura, Akiyuki Taruno, Minako Yagi, Kohei Yokota, Yukako Hayashi

**Affiliations:** 1 Graduate School of Agriculture, Kyoto University, Uji, Kyoto, Japan; 2 Department of Molecular Cell Physiology, Kyoto Prefectural University of Medicine, Kyoto, Kyoto, Japan; 3 PRESTO, JST, Kawaguchi, Saitama, Japan; Leibniz-Institute for Food Systems Biology at the TU Munich, GERMANY

## Abstract

Tolerance to bitter foods and its potentiation by repetitive exposure are commonly experienced and potentially underlie the consumption of bitter foods, but it remains unknown whether permissive and adaptive responses are general phenomena for bitter-tasting substances or specific to certain substances, and they have not been rigorously studied in mice. Here, we investigated the effects of prolonged exposure to a bitter compound on both recognition and rejection behaviors to the same compound in mice. Paired measurements of rejection (R_j_T) and apparent recognition (aR_c_T) thresholds were conducted using brief-access two-bottle choice tests before and after taste aversion conditioning, respectively. First, R_j_T was much higher than aR_c_T for the bitter amino acids _L_-tryptophan and _L_-isoleucine, which mice taste daily in their food, indicating strong acceptance of those familiar stimuli within the concentration range between R_j_T and aR_c_T. Next, we tested five other structurally dissimilar bitter compounds, to which mice were naive at the beginning of experiments: denatonium benzoate, quinine-HCl, caffeine, salicin, and epigallocatechin gallate. R_j_T was moderately higher than aR_c_T for all the compounds tested, indicating the presence of innate acceptance to these various, unfamiliar bitter stimuli in mice. Lastly, a 3-week forced exposure increased R_j_T for all the bitter compounds except salicin, demonstrating that mice acquire tolerance to a broad array of bitter compounds after long-term exposure to them. Although the underlying mechanisms remain to be determined, our studies provide behavioral evidence of innate and acquired tolerance to various bitter stimuli in mice, suggesting its generality among bitterants.

## Introduction

Bitter taste is generally considered a signal to avoid the ingestion of potentially toxic compounds; thus, the rejection of bitter-tasting foods is crucial for survival because many toxic compounds actually taste bitter. Bitter rejection is an innate response commonly seen in mammals, demonstrated by the fact that human infants and other mammals show hedonically negative facial expressions in response to bitter taste stimuli [1–4]. However, bitter sensation does not always evoke aversive responses. Acceptance of bitterness is commonly noted for certain foods such as vegetables, and it may be modulated by experience [5]. In the current study, we defined bitter tolerance as a reduction in the behavioral response to a bitter stimulus irrespective of whether bitterness is perceived, whereas bitter acceptance was defined as the consumption of a bitter solution despite the recognition of its bitterness.

It is clear that humans accept the bitterness of some foods and beverages. Also, the suggested self-medication of chimpanzees whereby they eat bitter plants may be a form of bitter acceptance. However, behavioral evidence of bitter acceptance in non-human experimental animals is still limited. Among a number of examples [6, 7], the work of Scott and Giza suggested acceptance of QHCl in rats. They quantified the preference for QHCl by brief-access gustometer tests with or without conditioned taste aversion (CTA) to the same compound. The preference curves for QHCl were significantly shifted to lower concentrations by CTA, revealing the QHCl acceptance range at low micromolar (10^−6^~10^−4^ M) concentrations. The detection of QHCl by rats at this concentration range is solely mediated by both the chorda tympani and glossopharyngeal nerves and the involvement of olfactory and trigeminal components is, if any, negligible [8]. Thus, rats accept the taste of QHCl at low concentrations even though they recognize its taste quality. However, it remains unknown whether acceptance is specific to QHCl or a general phenomenon to bitter-tasting substances, warranting further testing with various bitterants to elucidate the nature of bitter acceptance.

Aversive behavioral responses to bitterness are adaptive so that animals can minimize the number of false alarms derived from harmless but bitter foods. In the periphery, bitter sensation is mediated by G protein-coupled bitter taste receptors, TAS2Rs, expressed in the apical membrane of bitter-sensing taste bud cells. There are approximately 25 human *TAS2R*s and 35 murine *Tas2r*s [9–11]. Diverse ligand specificity and selectivity among TAS2Rs facilitate the detection of chemically diverse bitter compounds. Ligand-bound TAS2Rs become activated and cause cell excitation, leading to neurotransmitter release through CALHM1/CALHM3 channels towards the afferent taste nerves [12–15]. The gustatory neurons, including the chorda tympani, glossopharyngeal, and greater superficial petrosal nerves, synapse in the nucleus tractus solitarius of the brainstem. In the central nervous system, second-order neurons project to the ventroposterior medial nucleus of the thalamus and third-order neurons to the primary gustatory cortex for the recognition of taste quality, which further connects with higher brain regions including the amygdala [16] for decision-making on whether to accept or reject the food. Tolerance to bitter stimuli can be established by the modulation of any components in this bitter pathway. However, there are currently limited rodent behavioral data demonstrating the experience-dependent development of tolerance to bitter stimuli.

Using mice, the present study was conducted to assess whether acceptance is a general response to bitter compounds, and whether tolerance to them is evoked or potentiated by prolonged exposure. To address these questions, we estimated both rejection (R_j_T) and apparent recognition (aR_c_T) thresholds to various compounds before and after prolonged exposure to the same compound. We tested seven compounds that taste bitter to humans, including two bitter amino acids (_L_-tryptophan and _L_-isoleucine) that are contained in the laboratory mouse diet and are nutrients, and five examples of various classes of bitter chemical compounds (denatonium benzoate (DB), quinine hydrochloride (QHCl), caffeine (CAF), salicin (SAL), and epigallocatechin gallate (EGCG)) which are not contained in the daily diet and are not of any known nutritional value to mice.

## Materials and methods

### Ethics statement

All procedures for the care and treatment of animals were carried out according to the Japanese Act on the Welfare and Management of Animals and the Guidelines for the Proper Conduct of Animal Experiments issued by the Science Council of Japan. Studies were performed in accordance with protocol (26-4/27-4/28-4/29-4/30-4) approved by the Institutional Animal Care and Use Committee of Kyoto University. All efforts were made to minimize suffering and the number of animals used in this study. Mice were euthanized at the end of the study with CO_2_ followed by cervical dislocation.

### Animals

Female C57BL/6J SLc mice (5 weeks old) were purchased from Japan SLC (Hamamatsu, Japan). The mice were housed in standard plastic cages (3–4 per cage, 320 (W) × 220 (D) × 135 (H) mm, KN-600-T, NATSUME SEISAKUSHO, Tokyo, Japan) in the Kyoto University Animal Care Facility where the temperature and humidity were maintained at 23°C and 45–50%, respectively. The mice were maintained under a 12-h light/dark cycle with lights off at 10:00 and had *ad libitum* access to standard mouse chow and deionized water. After acclimation to the environment, the mice ranged in age from 6–7 weeks at the beginning of testing and were randomly divided into 12 groups, as described below. We limited this study to female mice to avoid errors that may be introduced by possible sex differences in preferences for tested compounds [17].

### Taste stimuli

_L_-Tryptophan, _L_-isoleucine, denatonium benzoate, quinine hydrochloride, caffeine, salicin, and sucrose were purchased from Sigma (St. Louis, MO, USA). Epigallocatechin gallate was provided by Taiyo Chemicals (Mie, Japan). All compounds were dissolved in deionized water to obtain the indicated concentrations.

The concentrations of bitter solutions used in this study are listed in Table 1. Pilot studies were conducted to determine the concentrations of each compound used in the two-bottle choice tests so that they spanned the range from indifference to marked avoidance [18, 19]. The concentrations used for conditioned taste aversion (CTA) were set near the aversion threshold in naive animals (NE group). The concentrations used during prolonged exposure periods were the same as those for CTA except for CAF. Lower concentrations of CAF were used during taste aversion conditioning and prolonged exposures because mice exposed to high concentrations of CAF for a long time exhibited abnormal behaviors such as disruption of the day-night cycle and agitated and highly active states in a pilot study.

**Table 1:** List of bitter compounds and concentrations (mM) used in this study.

Bitter compounds	2-bottle choice test	CTA	Long-term exposure
l-Tryptophan	1–60	50	N.A.
l-Isoleucine	1–300	200	N.A.
Denatonium benzoate	0.03–3	0.3	0.3
Quinine-HCl	0.001–1	0.03	0.03
Caffeine	0.3–50	5	3
Salicin	1–100	50	50
Epigallocatechin gallate	0.01–10	1	1

N.A., not applicable.

### Experimental schedules

**Fig 1:**
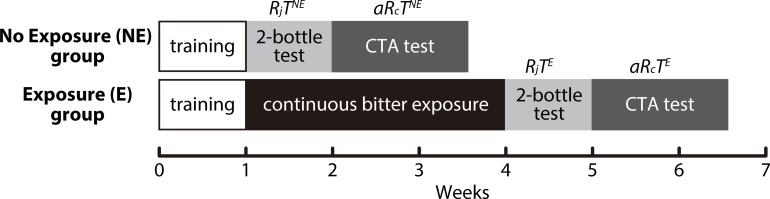
Typical experimental schedules for the measurement of rejection (R_j_T) and apparent recognition (aR_c_T) thresholds for bitter compounds. R_j_T^NE^ and aR_c_T^NE^ are defined as values measured without 3 weeks of a bitter exposure period (NE group), whereas R_j_T^E^ and aR_c_T^E^ as values measured after the continuous bitter exposure period (E group).

Except for _L_-tryptophan and _L_-isoleucine, paired measurements of R_j_T and aR_c_T to each of the other five bitter compounds were performed in two groups of mice: naive (no exposure, NE) and long-exposure (exposure, E) animals. Thus, there were 12 groups of mice with each mouse tested with only one compound in either the NE or E group: _L_-tryptophan (NE), _L_-isoleucine (NE), DB (NE and E), QHCl (NE and E), CAF (NE and E), SAL (NE and E), and EGCG (NE and E). Typical experimental schedules for NE and E groups are shown in Fig 1, although there were deviations of 2–3 days from the typical schedules depending on how quickly each mouse became trained to respond to two water bottles in a 10-minute period.

For the NE group, the mice were caged individually in standard plastic cages (125 (W) × 199 (D) × 113 (H) mm, CL-0113-1, CLEA JAPAN, Inc., Tokyo, Japan) and familiarized with having access to two drinking spouts. To achieve this, each mouse was water-deprived for 7 h from the onset of the dark period and then given access to two deionized water-filled drinking bottles for 10 min. We used custom-made plastic water bottles fabricated from Falcon 15-mL centrifuge tubes (#352096, Fisher Scientific, Hampton, NH, USA) and stainless steel sipper tubes with a stainless steel ball inside (TD-100, CLEA Japan, Inc.), with which we could avoid spillage during the tests. Each mouse was then returned to its home cage and given free access to food and water. This procedure was repeated every day until the mouse learned to drink water during the 10-min period. Once trained, the mouse was presented with two bottles: one bottle containing 100 mM sucrose and the other containing deionized water. At 1, 3, and 5 min, the positions of the bottles were switched to control for any side preferences. After 10 min, solution intakes were measured. This two-bottle choice test for 100 mM sucrose was repeated several times. Mice that reliably showed a preference for sucrose were subjected to the following experiments. During the next 8 days, preference ratios for the indicated concentrations of each bitter compound were measured by the two-bottle choice tests and R_j_T was estimated, as described below (Fig 1, R_j_T^NE^). Subsequently, the conditioned taste aversion tests consisting of aversion conditioning and the two-bottle choice tests for the same compound were performed to determine aR_c_T, as described below (Fig 1, aR_c_T^NE^).

The experimental schedule for the E group was similar to that for the NE group except that, immediately after the training period, there was a period of 3 weeks during which each mouse had *ad libitum* access to food and a solution of a test bitter compound instead of water. Mice in the same group were caged together in their home cage during the exposure period, and the total intake of the bitter solution was measured every 24 h. After this long-term exposure to the bitter stimulus, R_j_T and aR_c_T of the same compound were estimated in the same way as in the NE group (Fig 1, R_j_T^E^ and aR_c_T^E^). The mice in the NE and E groups were 7–12 weeks old (early adulthood) at the time of taste preference tests. During this period, taste preference and water intake of C57BL/6J mice are unaffected by age variations [19] (see also intake data in this study). Thus, changes in responses to bitter stimuli can be attributed to the 3-week exposure to a bitter stimulus.

### Brief-access two-bottle choice tests: Bitter rejection threshold (R_j_T)

This experiment was conducted to measure the threshold for behavioral rejection of the taste of a bitter compound (R_j_T). We used the protocol described in a previous study [20]. Mice were placed in standard plastic cages (125 (W) × 199 (D) × 113 (H) mm, CL-0113-1, CLEA JAPAN, Inc., Tokyo, Japan) and tested individually. We used the custom-made plastic water bottles described above to avoid spillage during the tests. One concentration of each test solution was tested per day and ascending concentrations were presented. Each mouse was water-deprived for 7 h from the onset of the dark period and then presented with two drinking bottles: one bottle contained a test solution with a known concentration of a bitter compound, and the other contained deionized water. At 1, 3, and 5 min, the positions of the bottles were switched to control for any side preferences, although the exact timing slightly varied (< 10 s) because we waited for the mice to stop drinking before switching the bottles in order to avoid interrupting their drinking (the positions of the bottles were switched when the mice were not drinking and frequently not even observing the bottles). Hereby, each bottle gets approximately equal number of drinking rather than equal time in each position. After 10 min, the mice were returned to their home cages and given *ad libitum* access to food and water until the next concentration was tested on the following day. There was no rest day during the concentration series. Solution intakes were measured based on weight differences of the drinking bottles before and after each test, and preference ratios were calculated as the ratio of the test solution intake to the total solution intake. R_j_T was defined as the lowest solution concentration at which the preference ratio was significantly lower than indifference (0.5).

### Conditioned taste aversion (CTA) tests: Apparent bitter recognition threshold (aR_c_T)

This experiment was designed to estimate the threshold for recognition of the taste of a bitter compound (R_c_T) by employing CTA. CTA is a form of learning whereby mice learn to associate a novel taste (conditioned stimulus, CS) with gastrointestinal malaise caused by LiCl injection (unconditioned stimulus, US) and consequently avoid drinking a solution with this specific taste not only at the concentration used as the CS but also at other concentrations within a certain range, known as the intensity generalization range. We defined the lower limit of the intensity generalization range as R_c_T. Unlike typical CTA experiments, we tested bitter compounds provoking innate aversiveness. If the CTA procedure decreases the threshold for behavioral rejection of a bitter compound (R_j_T > R_c_T), then the stimulus would be accepted in the concentration between R_j_T and R_c_T, suggesting that the mouse exhibits acceptance of the bitterness of this compound. It should be noted that mice experienced the CS before CTA in the preceding two-bottle choice tests and 3-week bitter-exposure period and, thus, were not totally naive to these bitter stimuli. Although such previous experiences of the CS may somewhat affect CTA development (through latent inhibition), they could not be avoided because, in some experiments, we aimed to estimate R_c_T of a stimulus after long-term exposure to the same stimulus. Thus, R_c_T estimated in this study is considered an "apparent" R_c_T (aR_c_T).

The mice were housed individually in standard plastic cages (125 (W) × 199 (D) × 113 (H) mm, CL-0113-1, CLEA JAPAN). Following 6 h of water deprivation from the onset of the dark period and brief licking of distilled water through a single drinking bottle for no more than 2 s, the mice were presented with a bitter solution as the CS. Immediately after having vigorously consumed the CS solution (the mice were very thirsty), the mice were given an intraperitoneal injection of 0.6 M LiCl (0.6 mL/kg B.W.) as the US. The CS-US interval was typically less than 30 s. Clear behavioral signs of visceral malaise (*e*.*g*., crouching and often vomiting) were used as criteria for the successful induction of illness. The mice were returned to their home cages and given free access to food and water, and this CS-US pairing was repeated the next day. One day later, we initiated brief-access two-bottle choice tests for an ascending series of concentrations of the same bitter compound and measured preference ratios as described above. Here, aR_c_T was defined as the lowest solution concentration for which the preference ratio was significantly lower than indifference (0.5). Although CTA lasts for more than a week under conditions whereby the avoidance of the CS is not life-threatening [21], water deprivation and post-conditioning CS experiences are known to facilitate the extinction of CTA [22–25]. Therefore, we employed a short period of water deprivation (7 instead of 23 h) and presented an ascending concentration series during the post-conditioning taste preference tests. We did not use complete avoidance of a CS as the criterion for a successful CTA because CTA does not necessarily cause a decrease in preference for the taste of a bitter compound if it already evokes maximal aversion in mice (avoidance for such compounds is unaffected by CTA). Therefore, we employed the CTA procedure that we know from experience is always maximally effective for other taste qualities (*i*.*e*., repeating the CS-US pairing twice, as described above), and assumed maximal aversive learning when estimating aR_c_T, although we cannot decisively rule out possible variations in the level of aversive learning.

### Data analysis

All statistical analyses were performed with SPSS statistics 21 (IBM, Armonk, New York, USA). Data were analyzed by mixed-design analysis of variance (ANOVA) with factors of Group (with and without prolonged exposure) and Concentrations, or two-way repeated measures ANOVA with factors of Treatment (before and after CTA) and Concentrations. All statistical values are shown in Tables. To determine R_j_T and aR_c_T, differences in preference ratios from indifference (0.5) were assessed by the one-sample *t*-test. All data are presented as the mean ± standard error of the mean. A *p*-value of < 0.05 was considered significant. The preference ratios for all tested concentrations within each group were fitted to a regression curve using the function: f(*x*) = 0.5/(1 + exp(*α*(log(*x*) − log(*β*)))), where *x* is the stimulus concentration, *α* is the slope, and *β* is the stimulus concentration at a preference ratio of 0.25.

## Results

### Preference to bitter amino acids

**Fig 2:**
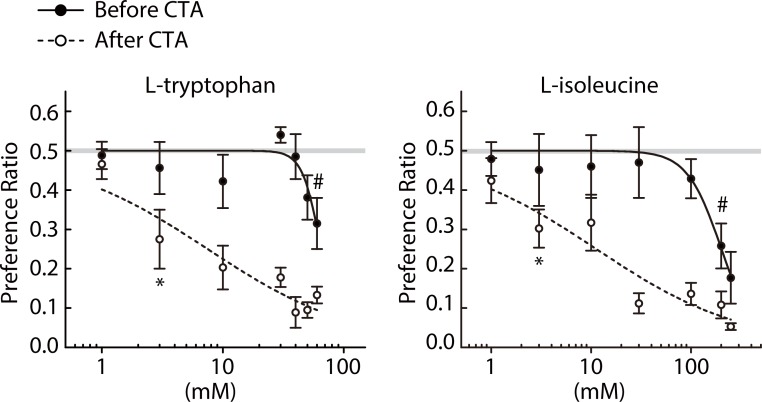
Two-bottle preference ratios for concentration series of _L_-tryptophan (n = 8) and _L_-isoleucine (n = 8) measured before (closed circles) and after (open circles) poisoning with LiCl. # and * indicate the lowest concentrations at which preference ratios fell significantly (*p* < 0.05) below indifference (0.5) before and after CTA, respectively. Solid and dashed lines represent curves fit to the average ratios before and after CTA, respectively. Gray lines represent the indifference level (0.5).

First, we investigated how mice respond to the tastes of two bitter amino acids, _L_-tryptophan and _L_-isoleucine. We performed the paired estimation of R_j_T and aR_c_T as described above following the NE group schedule in Fig 1. We chose these compounds because they are contained in the laboratory mouse diet and mice are conceivably familiar with their tastes. They are well-known bitter compounds for humans and TAS2R4 is a target of _L_-tryptophan, whereas the receptor for _L_-isoleucine is unknown. Fig 2 shows preference ratios for the indicated concentrations of the two compounds before and after CTA induced by intraperitoneal LiCl injection. There were significant differences in preference ratios between before and after CTA for _L_-tryptophan (Treatment × Concentration interaction, *F*(6,42) = 4.85, *p* = 0.001) but not for _L_-isoleucine (Treatment × Concentration interaction, *F*(6,42) = 1.462, *p* = 0.215) (Table 2). The thresholds for avoidance of both compounds markedly shifted to lower concentrations after CTA: the R_j_T and aR_c_T estimates for _L_-tryptophan were 60 and 3 mM and those for _L_-isoleucine were 200 and 3 mM, respectively (Fig 2 and Table 3).

**Table 2:** Summary of ANOVA results for preference scores.

Bitter compounds	Treatment	Concentration	Treatment X Concentration
l-Tryptophan	F(1,7) = 112.984, p<0.001	F(6,42) = 5.399, p<0.001	F(6,42) = 4.847, p = 0.001
l-Isoleucine	F(1,7) = 31.013, p = 0.001	F(6,42) = 9.089, p<0.001	F(6,42) = 1.462, p = 0.215
Denatonium benzoate	F(1,7) = 1.285, p = 0.294	F(6,42) = 30.189, p<0.001	F(6,42) = 0.456, p = 0.837
Quinine-HCl	F(1,6) = 5.081, p = 0.065	F(6,36) = 16.621, p<0.001	F(6,36) = 1.303, p = 0.281
Caffeine	F(1,7) = 44.315, p<0.001	F(7,49) = 41.251, p<0.001	F(7,49) = 0.924, p = 0.496
Salicin	F(1,6) = 20.463, p = 0.004	F(6,36) = 5.746, p<0.001	F(6,36) = 0.744, p = 0.618
Epigallocatechin gallate	F(1,7) = 5.906, p = 0.045	F(6,42) = 29.506, p<0.001	F(6,42) = 0.753, p = 0.611

**Fig 3:**
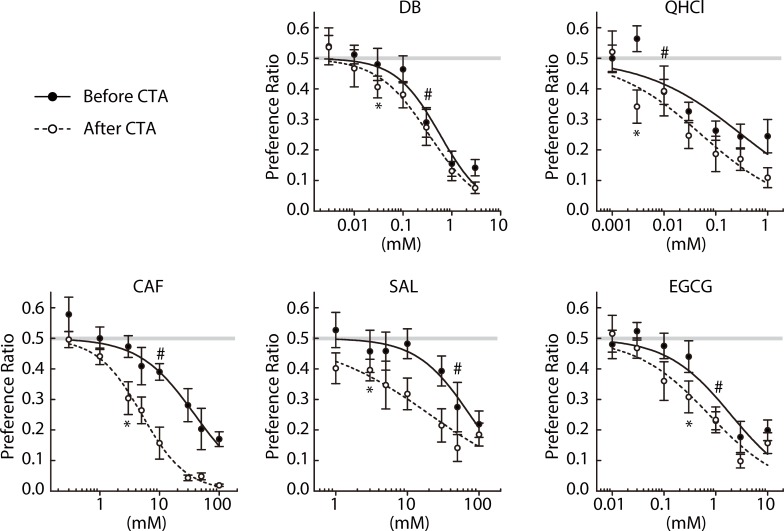
Two-bottle preference ratios for concentration series of denatonium benzoate (DB, n = 8), quinine hydrochloride (QHCl, n = 7), caffeine (CAF, n = 8), salicin (SAL, n = 8), and epigallocatechin gallate (EGCG, n = 8) measured before (closed circles) and after (open circles) poisoning with LiCl. # and * indicate the lowest concentrations at which preference ratios fell significantly (*p* < 0.05) below indifference (0.5) before and after CTA, respectively. Solid and dashed lines represent curves fit to the average ratios before and after CTA, respectively. Gray lines represent the indifference level (0.5).

Between-subject factor, Treatment (CTA); Within-subject factor, Concentration (Figs 2 and 3)

**Table 3:** Rejection and recognition thresholds for various bitter compounds.

Bitter compounds	R_j_T (mM)		aR_c_T (mM)	
	No Exposure	Exposure	No Exposure	Exposure
l-Tryptophan	60	N.D.	3	N.D.
l-Isoleucine	200	N.D.	3	N.D.
Denatonium benzoate	0.3	3	0.03	0.1
Quinine-HCl	0.01	0.1	0.003	0.03
Caffeine	10	50	3	3
Salicin	50	50	3	5
Epigallocatechin gallate	1	3	0.3	1

N.D., not determined.

### Preference to other bitter compounds

We then performed the paired estimation of R_j_T and aR_c_T for various classes of bitter compounds: DB, QHCl, CAF, SAL, and EGCG (NE group in Fig 1). Of note, they are not included in the laboratory mouse diet so mice may not have been familiar with their tastes before these experiments. Preference ratios for the indicated concentrations of the five compounds before and after CTA are plotted in Fig 3. There were no significant differences between before and after CTA in response to the five bitter stimuli (Treatment × Concentration interaction, *p* > 0.05, Table 2). R_j_T and aR_c_T are estimated and listed in Table 3. R_j_T was larger than aR_c_T for all unfamiliar bitter stimuli tested.

### Experience promotes bitter tolerance

**Fig 4:**
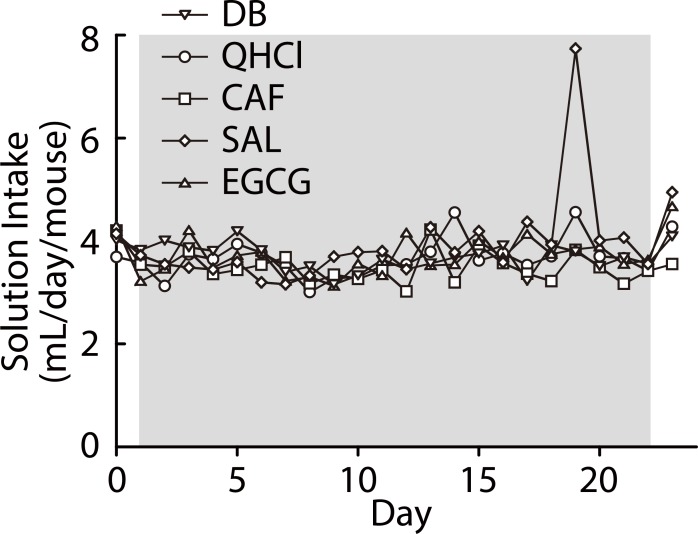
The average volume of daily solution intake per mouse during the prolonged bitter exposure period. The shadow indicates the period when water was replaced with a bitter solution.

**Fig 5:**
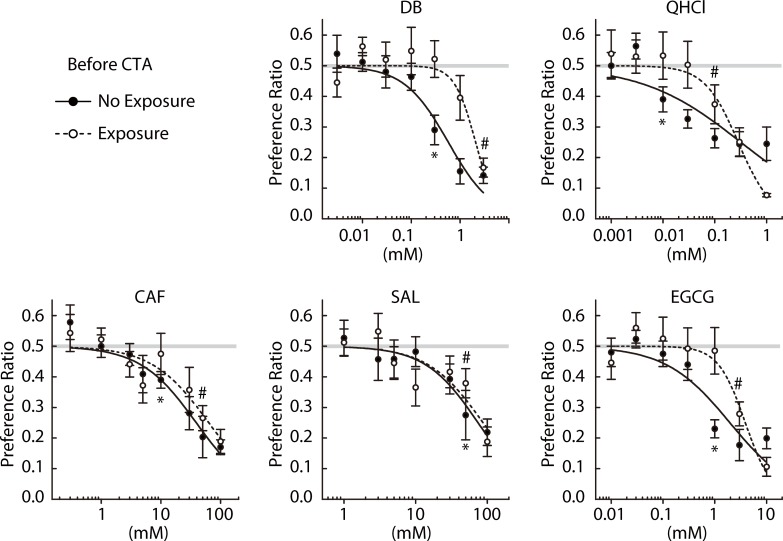
Two-bottle preference ratios for concentration series of DB (n = 8), QHCl (n = 7), CAF (n = 8), SAL (n = 8), and EGCG (n = 8) measured after a 3-week period of exposure to the same bitter compound (open circles) superimposed on those measured without the exposure period (closed circles). # and * indicate the lowest concentrations at which preference ratios fell significantly (*p* < 0.05) below indifference (0.5) before and after the long bitter exposure, respectively. Solid and dashed lines represent curves fit to the average ratios for the NE and E groups, respectively. Gray lines represent the indifference level (0.5).

We hypothesized that prolonged exposure to a bitter stimulus would attenuate aversive responses to that stimulus in mice. To test this hypothesis, we maintained mice for 3 weeks with the standard diet and a bitter solution instead of water, followed by the two-bottle choice tests to estimate R_j_T for the bitter stimulus used during the prolonged exposure period (E group in Fig 1). We tested the 5 unfamiliar bitter compounds presented in Fig 3. The average volume of daily solution intake per mouse during the exposure period is shown in Fig 4. Following a very small decrease in the intake volume at the start of the exposure, solution intake was stable over the period except for a high value observed in the SAL-exposed group, which may have been caused by spillage, with no increasing or decreasing tendencies for any of the compounds. Furthermore, the average intake volume over the period was comparable among compounds: DB, 3.67; QHCl, 3.65; CAF, 3.48; SAL, 3.71; EGCG, 3.64 (mL/day/mouse). There were no significant differences in weight gain during the 3-week exposure period between compounds: DB, 1.33 ± 0.57; QHCl, 1.28 ± 0.81; CAF, 0.62 ± 0.46; SAL, 1.21 ± 0.11; EGCG, 1.01 ± 0.64 (g) (*p* > 0.93, Tukey test). Also, compared with mice having free access to water (1.34 ± 0.44 g), the rate of weight gain was not significantly affected by the bitter exposure period (*p* > 0.88, Dunnet test). These data indicate that, during the prolonged exposure period, the levels of stable sensory experience of each compound were comparable among mice (note that a relatively lower stimulus level was used for CAF; See Methods) while maintaining water intake. Preference ratios for the indicated concentrations of each of the five compounds with and without prolonged bitter exposure are compared in Fig 5. There were significant differences between with and without prolonged exposure in response to DB, QHCl, and EGCG (Treatment × Concentration interaction, *F*(6,72) = 3.462, *p* = 0.005, *F*(6,72) = 2.46, *p* = 0.032, *F*(6,72) = 2.425, *p* = 0.034, respectively) but not to CAF and SAL (Treatment × Concentration interaction, *F*(7,91) = 0.534, *p* = 0.807, *F*(6,84) = 0.855, *p* = 0.532, respectively) (Table 4). We defined estimates for R_j_T with and without prolonged stimulus exposure as R_j_T^NE^ and R_j_T^E^, respectively. As seen in Fig 5 and Table 3, the prolonged exposure increased R_j_T for all compounds except SAL: that is, R_j_T^E^ was larger than R_j_T^NE^. The R_j_T^E^/R_j_T^NE^ ratios were: 10 (DB), 10 (QHCl), 5 (CAF), 1 (SAL), and 3 (EGCG).

**Table 4:** Summary of ANOVA results for preference scores.

Bitter compounds	Treatment	Concentration	Treatment X Concentration
Denatonium benzoate	F(1,12) = 4.33, p = 0.06	F(6,72) = 20.376, p<0.001	F(6,72) = 3.462, p = 0.005
Quinine-HCl	F(1,12) = 2.412, p = 0.146	F(6,72) = 14.507, p<0.001	F(6,72) = 2.46, p = 0.032
Caffeine	F(1,13) = 0.432, p = 0.522	F(7,91) = 14.514, p<0.001	F(6,84) = 0.855, p = 0.532
Salicin	F(1,14) = 0.059, p = 0.811	F(6,84) = 7.153, p<0.001	F(6,72) = 2.425, p = 0.034
Epigallocatechin gallate	F(1,12) = 5.769, p = 0.033	F(6,72) = 17.423, p<0.001	F(6,42) = 0.753, p = 0.611

Between-subject factor, Treatment (prolonged bitter exposure); Within-subject factor, Concentration (Fig 4)

### Effect of prolonged bitter exposure on aR_c_T

**Fig 6:**
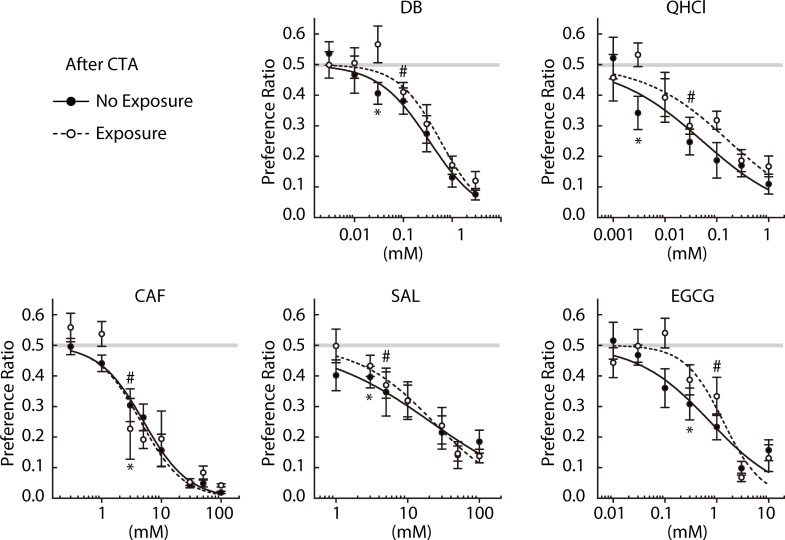
LiCl-conditioned avoidance of DB (n = 6), QHCl (n = 7), CAF (n = 7), SAL (n = 8), and EGCG (n = 6) in two-bottle choice tests after a 3-week period of exposure to the same bitter compound (open circles) superimposed on those measured without the exposure period (closed circles). # and * indicate the lowest concentrations at which preference ratios fell significantly (*p* < 0.05) below indifference (0.5) before and after the long bitter exposure, respectively. Solid and dashed lines represent curves fit to the average ratios for the NE and E groups, respectively. Gray lines represent the indifference level (0.5).

Finally, we estimated aR_c_T for each bitter compound used in Figs 3 and 5 after the 3-week exposure to the same stimulus using the two-bottle choice tests following CTA (E group in Fig 1). Preference ratios for each of the five compounds measured after CTA with and without prolonged bitter exposure are compared in Fig 6. There were no differences in preference ratios between with and without prolonged exposure (Treatment × Concentration interaction, *p* > 0.05, Table 5). We defined aR_c_T estimates with and without long-term exposure as aR_c_T^NE^ and aR_c_T^E^, respectively. As indicated in Fig 6 and Table 3, modest increases in aR_c_T were noted after 3-week continuous exposure to the bitter stimuli: The aR_c_T^E^/aR_c_T^NE^ ratios were: 3.3 (DB), 10 (QHCl), 1 (CAF), 1.7 (SAL), and 3.3 (EGCG).

**Table 5:** Summary of ANOVA results for preference scores.

Bitter compounds	Treatment	Concentration	Treatment X Concentration
Denatonium benzoate	F(1,12) = 1.872, p = 0.196	F(6,72) = 29.889, p<0.001	F(6,72) = 0.88, p = 0.47
Quinine-HCl	F(1,12) = 4.306, p = 0.06	F(6,72) = 13.26, p<0.001	F(6,72) = 1.329, p = 0.255
Caffeine	F(1,13) = 0.811, p = 0.384	F(7,91) = 36.086, p<0.001	F(7,91) = 0.903, p = 0.508
Salicin	F(1,12) = 0.458, p = 0.512	F(6,72) = 12.715, p<0.001	F(6,72) = 0.407, p = 0.872
Epigallocatechin gallate	F(1,12) = 2.868, p = 0.116	F(6,72) = 22.164, p<0.001	F(6,72) = 1.630, p = 0.151

Between-subject factor, Treatment (prolonged bitter exposure); Within-subject factor, Concentration (Fig 5)

## Discussion

Two questions were addressed in mice: (1) whether there is an acceptable range of the bitter stimulus intensity for various bitter compounds, and (2) whether prolonged experience of a bitter stimulus leads to the attenuation of aversive responses to that stimulus. We measured R_j_T and aR_c_T of a bitter compound before or after a 3-week forced exposure to a solution containing the same compound instead of water, and we tested seven compounds tasting bitter to humans, five of which except _L_-tryptophan and _L_-isoleucine have been demonstrated to elicit aversion in mice. The 10-min brief-access two-bottle tests were performed to minimize post-ingestive effects. The concentrations for CTA and exposure periods (Table 1) were appropriate based on observations of significant shifts of R_j_T and aR_c_T estimates (Figs 2, 3, 5 and 6 and Table 3).

Acceptance of QHCl in rats was suggested in a previous study [7]. Whereas the authors of the study tested all concentrations in a single test session and measured the numbers of licks, we tested one concentration in a session and measured the intake volume. Using a mouse model, we expanded the number of bitter stimuli with which bitter acceptance was tested, and tested seven substances including QHCl. Larger R_j_T than aR_c_T values were observed for all compounds tested (Figs 2 and 3 and Table 3). R_j_T represents the lowest concentration for which an unconditioned mouse rejects a bitter compound, and aR_c_T represents the lowest concentration for which the same mouse rejects it after aversion conditioning to a known concentration of the same compound (the intensity generalization threshold). Although we did not intensively examine how accurately aR_c_T obtained for each of the seven bitterants reflects the actual R_c_T that corresponds to the sensitivity of peripheral taste mechanisms to the stimulus [26, 27], a gap between R_j_T and aR_c_T revealed by CTA clearly indicates the acceptance of the bitterant within this concentration range. Thus, the seven structurally and chemically diverse bitter substances are all accepted at low concentrations by mice, supporting the previous observations in rats and further indicating that bitter acceptance is a general response to broad bitter stimuli. Of note, the apparent acceptance ranges roughly estimated by the R_j_T/aR_c_T ratios for _L_-tryptophan and _L_-isoleucine (20 and 66.7, respectively) are appreciably higher than those for the other unfamiliar bitter compounds (10, 3.3, 3.3, 16.7, and 3.3 for DB, QHCl, CAF, SAL, and EGCG, respectively). These observations suggest that the degree of acceptance may be modified by previous experience.

Experience-dependent attenuation of gustatory responses has been studied more rigorously in herbivorous insects [28, 29] than in mammalian models. However, it was reported that in guinea pigs, a 3-week exposure to sucrose octaacetate, a bitter substance, in the newborn period caused a reversible loss of aversive response to this substance via unknown mechanisms, providing an example in rodents [30]. Torregrossa and her team provided another example whereby rats administered a tannin-containing diet found the tannin solution less aversive than control rats [31]. We investigated whether other bitter compounds also induce similar tolerance in mice after prolonged exposure. Our analyses of DB, QHCl, CAF, SAL, and EGCG revealed that, after a 3-week forced exposure to a solution of any one of the five bitter compounds except for SAL, mice responded to the same solution as if it was of a lower concentration, as demonstrated by an increase in R_j_T (R_j_T^E^/R_j_T^NE^ > 1). Regarding SAL, it remains to be determined whether the sensation evoked by the concentration used during the exposure period was insufficient to induce tolerance to SAL or that mice were already maximally tolerant of the bitterness of SAL, as suggested by the broadest acceptance range (R_j_T^NE^/aR_c_T^NE^ ratio = 16), so that experience could not further potentiate tolerance to SAL. Nevertheless, our results clearly demonstrate that tolerance can be acquired by experience for many, if not all, bitter stimuli.

Mechanisms underlying acquired bitter tolerance remain largely unexplored. In *Manduca sexta* caterpillars, 24-h exposure to CAF reduced their aversive behavioral response to it, which is mediated by the desensitization of taste cells [28]. However, experience-dependent desensitization of the responsiveness of taste cells has not been reported in mammals. Instead, there is accumulating evidence of the interaction of saliva proteins and taste experience. The saliva of healthy adults contains hundreds of different proteins [32], and the saliva proteome pattern has been shown to be an indicator of bitter sensitivity and acceptance [33, 34] and be altered by bitter stimuli [35]. In rats, there is an indisputable role of saliva proteins in the development of tolerance to tannins, bitter and astringent substances contained in tea and red wine. Rats innately avoid tannins, but dietary exposure to them upregulates salivary protein expression, which makes tannins less aversive [31]. It has been suggested that elevated salivary proteins bind to tannic acid and lower its free concentration [36]. A recent report demonstrated the ability of QHCl to upregulate salivary proteins in rats, suggesting that the adaptive salivary response to a bitter stimulus is not specific to tannins, but may be a more general phenomenon [37]. Importantly, the same study revealed that saliva proteins suppress the gustatory nerve responses to QHCl but not NaCl [37]. Thus, the experience-dependent alteration of the salivary proteome pattern may be a mechanism underlying sensory adaptation to bitter stimuli. Our observations may provide some clues regarding peripheral perception. The prolonged exposure period caused modest increases in aR_c_T (Fig 6). As mentioned above, however, aR_c_T may not be an accurate measure of R_c_T, which reflects peripheral taste sensitivity. Especially, given the much longer pre-exposure to the CS before CTA, one would expect a larger overestimation of aR_c_T^E^ compared with aR_c_T^NE^, so our analyses of aR_c_T may have weaknesses regarding elucidating the effects of sensory adaptation on acquired bitter tolerance. However, it should also be noted that a steeper slope at intermediate concentrations in the concentration-response function was proposed to be indicative of sensory inhibition by salivary proteins, as seen in rats for QHCl and tannins [31, 37]. In Fig 5, the concentration-response functions were noticeably steepened by chronic exposure to DB, QHCl, and EGCG. The changes in the two independent measures related to peripheral perception, aR_c_T and the slope of the concentration-response curve, suggest the contribution of sensory adaptation to the acquired tolerance to these compounds, warranting further studies exploring the effects of chronic exposure to bitter stimuli on the responsiveness of taste cells or gustatory nerves and the possible involvement of the saliva proteome.

Another possible mechanism of acquired bitter tolerance is habituation, a central mechanism [38]. Habituation is a form of non-associative learning, in which animals learn to ignore a familiar, biologically irrelevant, stimulus after repeated presentations with no consequences. Although our results do not directly evaluate habituation, an expansion of the apparent acceptance range calculated as the R_j_T/aR_c_T ratio, may, if weakly, correlate with the degree of habituation. In other words, a mouse can tolerate a stronger perceived bitter sensation through habituation. The ratio of R_j_T^E^/aR_c_T^E^ to R_j_T^NE^/aR_c_T^NE^ varied among the bitter compounds tested in this study: 3 (DB), 1 (QHCl), 5 (CAF), 0.6 (SAL), 0.9 (EGCG). The ratio was greater than unity for DB and CAF, implying the contribution of habituation to the acquired tolerance. Taken together, both cognitive and sensory evaluations should be considered in future work to fully understand the mechanisms behind the acquisition of bitter tolerance.

Physiological systems to discriminate between different bitter stimuli would be invaluable for animals to discriminate harmless and even beneficial bitter foods from harmful ones. Our data indicate that acquired tolerance is a phenomenon common to a broad array of bitter compounds, but they also suggest the presence of subtle differences in expression as described above, which may possibly help animals to select which bitter compounds to ingest. What quality induced these differences? Because agonist profiles are distinct among TAS2Rs [18], the set of bitter receptors activated by a bitterant and their distribution within taste buds may contribute. However, it remains controversial whether bitter-tasting compounds are qualitatively discriminable. In this regard, there is conflicting genetic [9, 39, 40] and cell physiological [41, 42] evidence. Human psychophysical studies have proposed multiple separate, yet perhaps overlapping, bitter transduction pathways [43, 44], although these human studies did not address issues of bitterness quality. Despite all of the above, rats cannot perceptually distinguish two structurally dissimilar bitter compounds, QHCl and DB [45]; thus, at least some bitter compounds share indiscriminative bitterness qualities. Our preliminary experiments also indicate that CTA manipulation of the response to QHCl decreases the avoidance thresholds of CAF, SAL, and EGCG, supporting the presence of a shared bitter taste quality among various classes of bitter substances. In addition, side tastes, associated odors, somatosensory sensations, and post-ingestive physiological effects might also be involved in the discrimination of bitter foods in the real world. For example, EGCG evokes an astringent sensation mediated by the trigeminal nerve [46]. The interpretation of our CAF data requires extra caution. CAF is well known as a psychomotor stimulant, and because of its diverse positive post-ingestive effects, it is widely consumed in drinks such as coffee and tea and used as medications [47, 48]. Indeed, in rats, lower doses of intraperitoneal CAF injection are rewarding, but higher doses can induce conditioned taste aversion due to their harmful effects [49], demonstrating dose-dependent biphasic non-gustatory effects. Acquired tolerance to non-gustatory CAF effects must also be kept in mind during chronic treatments [48]. Furthermore, we had to use lower stimulus intensities in the CTA and 3-week exposure period for CAF to minimize the symptoms of caffeinism, which may have affected conditioned learning and tolerance acquisition compared with the other compounds. Thus, the CAF and other data cannot be directly compared. Overall, our study does not allow us to assess the involvement of these non-taste qualities. It is now necessary to elucidate the factors that can influence tolerance to bitter stimuli. Those factors, however, might affect bitter tolerance differently in humans and mice just as human and mouse orthologous TAS2Rs have distinct agonist profiles [18]. Finally, as this study tested only a small number of chemically and structurally diverse compounds, more compounds need to be tested to gain insight into what characteristics of bitterants make observed differences.

Our results reveal that bitter acceptance is a general response to low concentrations of bitter substances, and tolerance to bitter stimuli can be induced by long-term presentation, although the underlying mechanisms remain unknown. Our study also provides preference data for two amino acids, _L_-tryptophan and _L_-isoleucine, that have not been tested in mice, suggesting that mice find them as bitter as humans do because they evoked aversive behavioral responses (Fig 2) and, similarly to many other known bitter stimuli, stronger nerve responses in the glossopharyngeal nerve than in the chorda tympani nerve have been reported in mice [50]. Because this study was limited to female mice, further studies are necessary to extend these findings to male mice. This work is an initial step toward understanding how bitter tolerance develops in mice, and possibly mammals in general.
